# Detection of varicella-zoster virus from cerebrospinal fluid using advanced fragment analysis in a child with encephalitis: a case report

**DOI:** 10.1186/s12879-019-3986-3

**Published:** 2019-04-25

**Authors:** Yu-xin Song, Ye Li, Yong-mei Jiang, Ting Liu

**Affiliations:** 1 Department of Laboratory Medicine, West China Second University Hospital, Sichuan University, No. 20, Section 3, Renmin Road South, Chengdu, Sichuan 610041 People’s Republic of China; 2 Key Laboratory of Obstetric and Gynecologic and Pediatric Diseases and Birth Defects of Ministry of Education, Sichuan University, Chengdu, China; 3 State Key Laboratory of Biotherapy and Cancer Center/National Collaborative Innovation Center for Biotherapy, Sichuan University, Chengdu, China

**Keywords:** VZV encephalitis, Advanced fragment analysis, Laboratory medicine

## Abstract

**Background:**

Varicella zoster virus (VZV) encephalitis is an infectious inflammatory disease of brain that can cause irreversible mental damage without timely treatment. In fact, many viruses can cause encephalitis, and the viral loads in cerebrospinal fluid (CSF) in the early stage of the disease are usually too low to be detected. Here we report a case of VZV encephalitis diagnosed by advanced fragment analysis (AFA), which could potentially to contribute to early diagnosis of VZV central nervous system (CNS) infections with a small volume of CSF samples.

**Case presentation:**

A 10-year-old boy was admitted to the hospital with obvious neurological symptoms of headache, dizziness and vomiting for one day. Physical examination showed left facial paralysis. Complete blood count (CBC) test only showed an unspecific inflammation, and the culture of cerebrospinal fluid and microscopic staining examination were all negative. AFA was performed to screen the common 18 encephalitis related pathogens in CSF. Obvious VZV DNA fragments were observed by capillary electrophoresis at 160 nt, suggesting the existence of VZV CNS infection in children. The results were consistence with real-time quantitative PCR and concomitant symptoms in the acute stage of the disease.

**Conclusions:**

We report a case of acute VZV encephalitis in a child without obvious skin manifestations, which was rapidly diagnosed by AFA. Overall, we would recommend the use of AFA analysis as the rapid screening system for the identification and differentiation of encephalitis pathogens in children.

## Background

Encephalitis is a kind of inflammatory disease of brain that is caused especially by infection with bacterial, virus and fungi [1–3]. Clinically, the encephalitis can seriously affect the central nervous system (CNS) and therefore leading life-threatening complications [4–6]. Among all the infectious agents of encephalitis, the viruses constitute majority of the cases. There are multiple viruses that can result in viral encephalitis including herpes simplex viruses (HSV), varicella-zoster virus (VZV), epstein-barr virus (EBV), and human herpesvirus-6 (HHV-6) [7–9]. In particular, viruses are difficult to be cultured in clinic, and the timely treatment is of great importance of the prognosis of encephalitis. Therefore, it is necessary to develop new diagnostic methods to quickly diagnose infectious encephalitis.

Varicella-zoster virus (VZV) is an important α-herpesvirus which can lifelong remain latent in trigeminal and dorsal root ganglia, and therefore might lead various neurologic diseases such as meningitis, encephalitis, myelitis, meningoencephalitis, cranial neuropathy, and peripheral neuropathy [10, 11]. In terms of the age of onset, the incidence of VZV infection is becoming younger-age trend. Accumulating studies shown that most cases of chicken pox (varicella) are occurred in the children aged between 5 and 14 years old. Primary VZV infection in healthy children is generally a mild, self-limiting disease which typically presents as a vesicular rash. However, in immunocompromised individuals and susceptible adults, particularly pregnant women, the infection can be more serious and even threatening the life [9, 12]. Once this general chickenpox develops to encephalitis, it may cause irreversible damage to both the brain and the body, such as mental retardation, stroke, giant cell arteritis and granulomatous aortitis, resulting in persistent inflammation and pathological vascular remodeling [9, 13]. Generally, the VZV infection leads to typical signs of chicken pox (varicella) that is very visible from the surface of the skin, and later possible to cause numbers of sub-acute, acute and chronic neurological complications [14]. However sometimes, the VZV may be also a latent infection for decades without obvious clinical symptoms [15–17]. Therefore, it is difficult for some patients to diagnose VZV infection based only on clinical manifestations.

Common diagnostic methods of VZV include general medical tests and more accurate pathogen tests [15, 18]. As we all known, the general medical tests include blood routine test, cerebrospinal fluid cytology (CSFC) test and biochemical test. These tests can indicate viral encephalitis, but it is not certain which virus causes the infection. In addition, the pathogen tests include virology test, antibody detection and molecular biology test. However, as far as we know, it is very difficult to grow viruses in medical laboratories, mainly because of the high requirements for virus culture. Besides, the culture results were closely related to the virus concentration of CSF. What’s more, the need of CSF volume of culturing is large, usually 1-2 ml, and it is not easy to collect enough CSF in children for those tests as well. Therefore, there is an urgent need for a new technology for the rapid and simple diagnosis of VZV viral encephalitis.

At present with the development of molecular diagnostic technology, the application of molecular techniques such as advanced fragment analysis (AFA) could be a useful method of identifying and confirming the existence of VZV infection in clinical specimens, especially in the CSF. Here, we report on an indigenous case of encephalitis in a 10-year-old male child with a recessive VZV infection, which rapidly diagnosed by AFA method.

## Case presentation

A 10-year-old boy (weight, 32.1 kg; height, 138.7 cm) in Sichuan province was admitted to the hospital with neurological symptoms of headache, dizziness and vomiting for one day. Physical examination showed left facial paralysis and no rash. His temperature is 36.5 °C, breath is 21 times per minute, pulse is 109 beats per minute, and the blood pressure is 103/72 mmHg. Additionally, he had no previous history of infection for these days.

According to the general medical tests, we found the percentage of neutrophil in peripheral blood increased to a number of 85.2% (reference range 23.6–75%), which suggests the presence of inflammation. In addition, we punctured cerebrospinal fluid for accurate examination. The CSF biochemical examination showed that the protein (136.8 mg/L), glucose (3.85 mmol/L), chloride (127 mmol/L) and lactate dehydrogenase (12 U/L) were all in the reference range. The cerebrospinal fluid cytology (CSFC) tests showed that the CSF was colorless and transparent, containing 25 × 10^6^/L nucleated cells and 13× 10^6^/L erythrocytes. Among the nucleated cells, lymphocytes and monocytes account for 98 and 2% respectively. These results indicated the presence of infectious encephalitis. However, the ink staining, gram staining and acid fast staining of CSF were all negative. Besides the bacteria culture of CSF for 5 days was negative as well. Therefore, the child was suspected to have acute viral encephalitis.

The pathogenesis of acute viral encephalitis involves a variety of viruses. Due to the small volume of CSF in children, the diagnosis of encephalitis related pathogens in CSF is very difficult, especially when the clinical symptoms are not obvious. In this case, we firstly test the IgM and IgG antibodies of herpes simplex virus (HSV), because HSV is the main virus that causes viral encephalitis [19, 20]. The results of electrochemical luminescence test showed that both antibodies were negative. Therefore, HSV infection was excluded and further experiments were needed to determine the etiology of encephalitis.

**Fig. 1:**
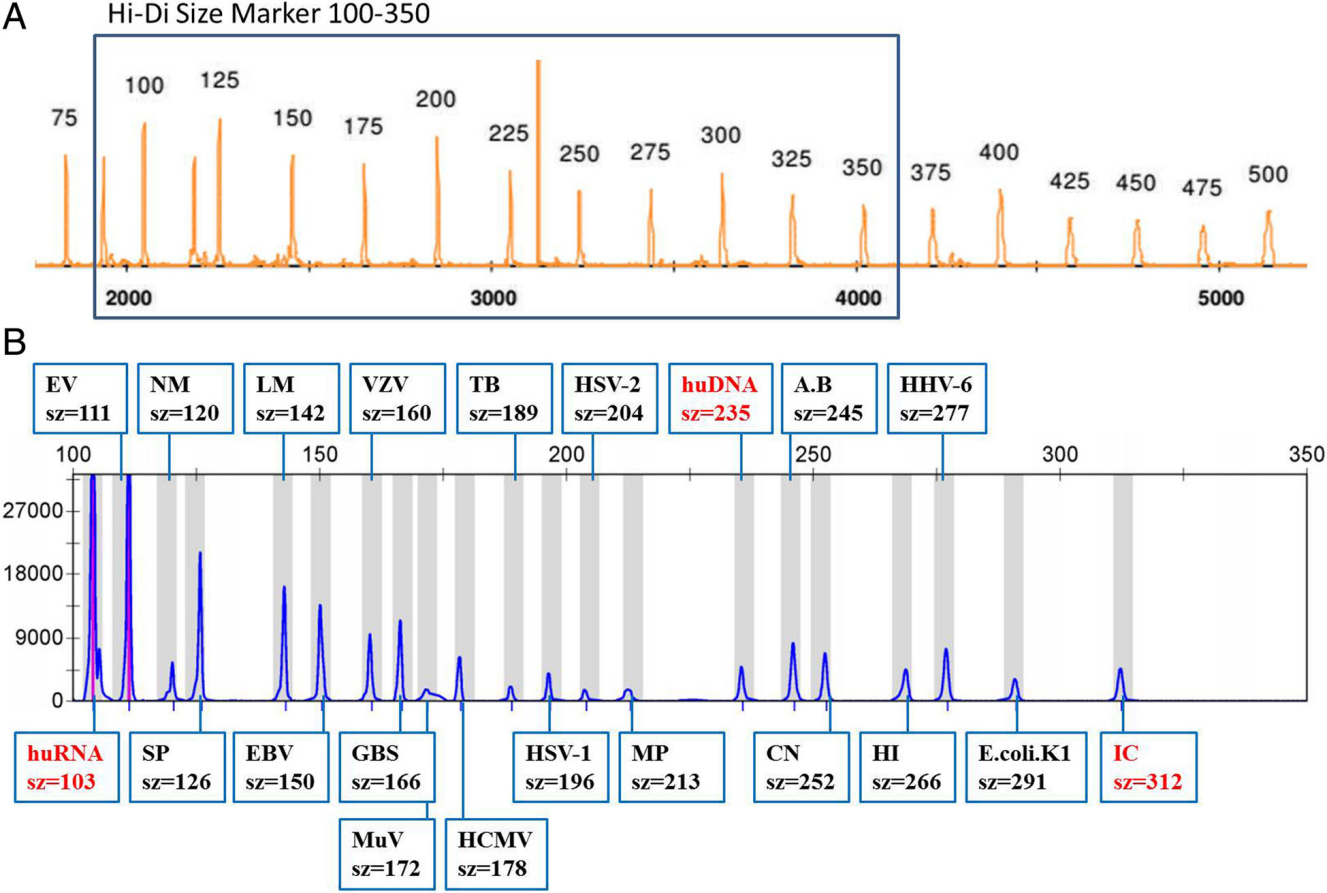
Application of advanced fragment analysis (AFA) in screening of encephalitis related pathogens from cerebrospinal fluid (CSF). 300 μl cerebrospinal fluids were used for fragment analysis. After multiplex PCR, the fragments with particular size were separated with capillary electrophoresis, and each fragment of a specific size represents a specific bacterial or virus infection of interest. **a** Hi-Di size maker determines the size of fragments by conducting capillary electrophoresis together with samples. **b** The capillary electrophoresis peaks of representative positive control sample. Different sizes of PCR fragments represent different pathogens

Advanced fragment analysis (AFA) is a molecular technique, which provides an alternative method for high-throughput multiplexed quantitative pathogens related gene expression. AFA integrates multiplex PCR and capillary electrophoresis to offer a simple and effective way to analyze dozens of specific genes in a single tube [21]. In this experiment, 300 μl CSF was used for total DNA and RNA extraction (ZD Biotech, China). 18 kinds of common encephalitis related pathogens can be detected at same time, including enterovirus (EV), *Cryptococcus neoformans* (CN), neisseria meningitidis (NM), *Streptococcus pneumoniae* (SP), epstein-barr virus (EBV), varicella-zoster virus (VZV), cytomegalovirus (HCMV), tuberculosis (TB), herpes simplex virus-1 (HSV-1), herpes simplex virus-2 (HSV-2), mycoplasma pneumoniae (MP), *Escherichia coli* (E.Coli), *Listeria monocytogenes* (LM), group B streptococcal (GBS), mumps virus (MuV), human herpes virus-6 (HHV-6), *Haemophilus influenzae* (HI) and acinetobacter baumannii (A.B) (Fig. 1). Briefly, for the multiplex PCR, we put 4.5 μl PCR Mix, 0.5 μl enzyme and 5 μl nucleic acid together (Health Gene Technologies, China). All reactions were run on an ABI 7500 real-time PCR system (Life Technologies, CA, USA) using the following cycling parameters: 25 °C for 5 min; 50 °C for 30 min; 95 °C for 15 min; 6 cycles of 94 °C for 30 s, 65–60 °C for 30 s and 72 °C for 60 s; 29 cycles of 94 °C for 30 s, 60 °C for 30 s and 72 °C for 60 s; and a final elongation step of 72 °C for 15 min. For capillary electrophoresis, the SIZE-500 Plus dissolved in Hi-Di formamide was used as the DNA size maker. All reactions were run on 3500Dx (Life Technologies, CA, USA) and the cutoff value is set to 500. Different sizes of PCR fragments represent different pathogens. With the AFA technology, the turn-around time is greatly reduced to 4 h, which is conductive to rapid diagnosis, timely treatment and prognosis of the encephalitis.

**Fig. 2:**
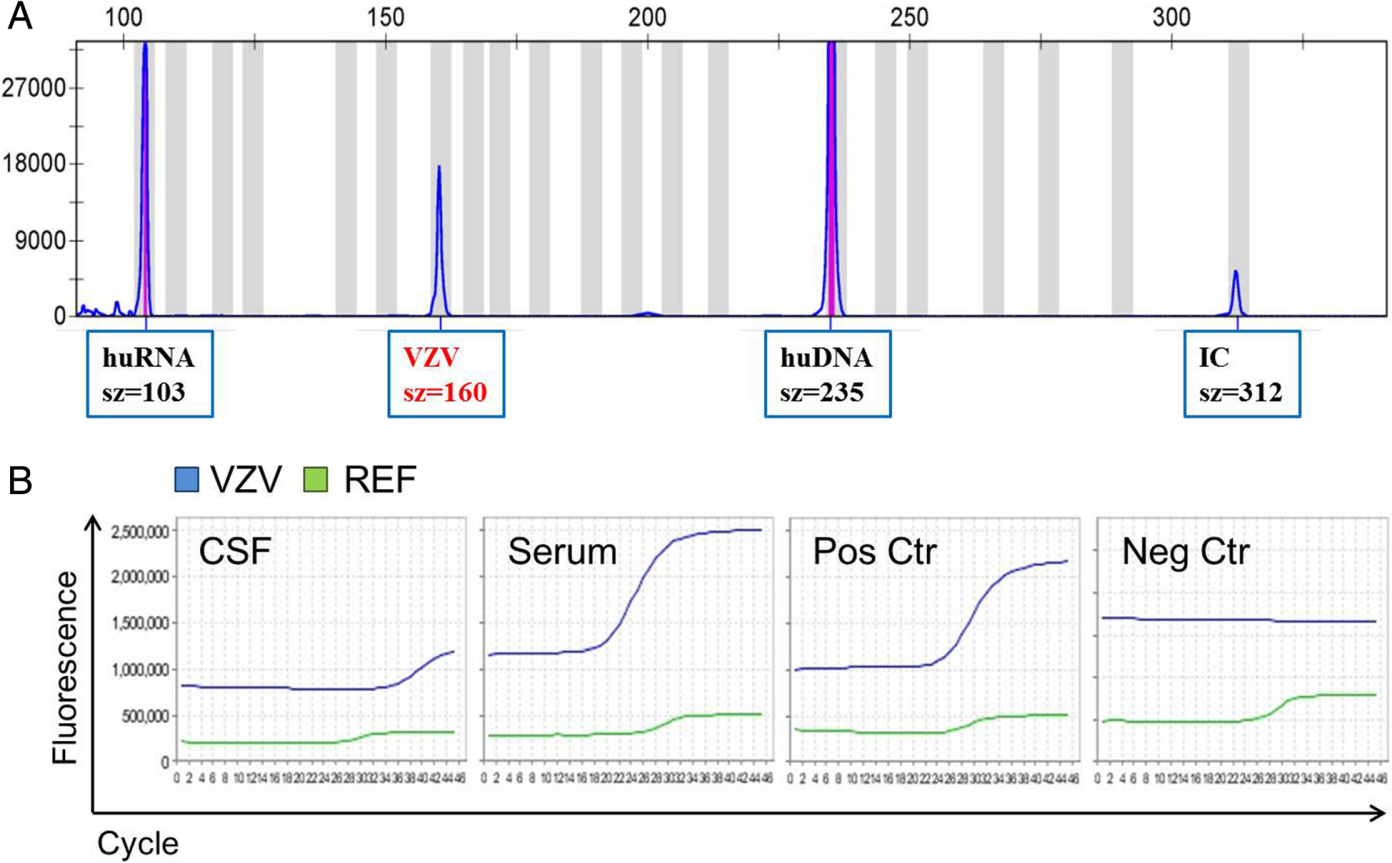
Detection of varicella-zoster virus (VZV) in cerebrospinal fluid (CSF) using advanced fragment analysis (AFA). **a** Obvious VZV DNA fragments were observed by capillary electrophoresis at 160 bp, suggesting the existence of VZV central nervous system infection in children. **b** The results were confirmed by detecting VZV DNA in both CSF and serum via real-time quantitative PCR. Similar results were observed in two independent experiments

Subsequent advanced fragment analysis of the pathogens gene suggested the presence of VZV infection as we found a fragment peak at 160 bp of capillary electrophoresis (Fig. 2a). It is worth mentioning that the child initially presented with left facial paralysis, but did not appear chicken pox. We know that primary infection with the VZV usually manifests with chickenpox, however reactivation of VZV can present with different clinical manifestations. Reactivation of VZV from the geniculate ganglion, the nucleus of the sensory root of the facial nerve, can cause peripheral facial weakness as well as rash around the ear, known as Ramsay Hunt syndrome [22]. Therefore, even if the patient’s parents denied that the child has a history of chickenpox infection, the child may have latent VZV infection in the genicular ganglion in the past. To verify the above results, on subsequent real-time fluorescence quantitative PCR was used to detect the VZV DNA in both CSF and serum samples using commercial kits at the time of acute disease (Sansure Biotech, China). Briefly, 10 μl of extracted DNA was amplified with PCR super mix reagent in 25 μl reaction volume for 35 cycles using the Life PCR System ABI 7500 (Life Technologies, CA, USA). Results were considered positive when a clear amplification curve of the expected site was obtained (Ct < 40). Consistent with the previous results, VZV DNA was detected in both CSF and serum (Fig. 2b). As shown in Fig. 2b, peripheral blood showed high viral load during the acute stage of the disease, though the viral load is less in the cerebrospinal fluid (CSF). Therefore, it is proved that the AFA technology is efficient and fast in detection the etiology of encephalitis.

Taken together, this report outlines the potential difficulties in differentiating and diagnoses pathogens in patients with encephalitis, which has particularly important implications for patient segregation and infection treatment. Our study might be of great importance to explore the application of the AFA technology in clinical diagnosis of encephalitis related pathogens in CSF. Still now, only a few isolated cases of VZV associated encephalitis have been reported.

## Discussion and conclusions

Body fluids, especially the CSF from child, present a diagnostic challenge due to the small sample volume and paucity of slow growing or uncultivable organisms those results in lower test sensitivity [23, 24]. This study was carried out with the objectives to recommend a rapid screening system of identifying common encephalitis pathogens in CSF by means of advanced fragment analysis (AFA). And later we confirmed the result from conventional method of culture, PCR and clinical manifestation.

The advanced fragment analysis (AFA) is a kind of molecular biological diagnostic technology, which combined multiplex polymerase chain reaction (PCR) with capillary electrophoresis (CE) together. Usually, the experimental process consists of three parts. Firstly, we extract total nucleic acid of pathogenic microorganisms including both DNA and RNA from samples. Secondly, we amplified the specific region of nucleic acid in pathogenic microorganisms by multiple PCR. Therefore we can obtain several amplification products with dedicated fragment sizes (typically approximately 100–350 Da). In this study, we have 18 pairs of specific primers in the reaction fluid, which indicates that we can use the single experiment to screen for 18 common encephalitis related pathogenic microorganisms simultaneously. Last, we separate the fragments with capillary electrophoresis, and each fragment of a specific size represents a specific bacterial or virus infection of interest.

As a new type of molecular technique, the key feature of fragment analysis is that multiple microbial infections can be detected at one time using a small volume of sample (300 μl) within approximately 4 h. While in terms of the traditional testing technique, 3–5 ml CSF was needed in pathogenic microorganisms culturing test for more than 2 weeks. Furthermore, thanks to the using of multiple PCR, the amount of nucleic acid which needed in the experiment is far more less than that in traditional PCR technology. On this occasion, the fragment analysis can be also applied to samples with low abundant or low nucleic acid concentration, including cerebrospinal fluid (CSF), feces, and pharynx swabs and so on. This greatly enriched the type of specimens that we can test, and enabled the early diagnosis of a small number of pathogenic microbial infections.

In a word, the application of AFA in screening for encephalitis related pathogens in child has many advantages. The whole experiment process is fast, the operation is simple, the sensitivity is high, the species of testing pathogenic microorganism is abundant, and the amount of sample needed is less. All these characteristics make AFA technique to be one of the excellent laboratory methods for detecting bacterial and viral encephalitis in child. Overall, we would recommend the use of AFA analysis as the rapid screening system for the identification and differentiation of encephalitis pathogens in child.

## Abbreviations

A.B: acinetobacter baumannii
AFA: Advanced fragment analysis
CBC: Complete blood count
CE: Capillary electrophoresis
CN: *Cryptococcus neoformans*
CNS: Central nervous system
CSF: Cerebrospinal fluid
CSFC: Cerebrospinal fluid cytology
E.Coli: *Escherichia coli*
EBV: Epstein-barr virus
EV: Enterovirus
GBS: Group B streptococcal
HCMV: Cytomegalovirus
HHV-6: Human herpes virus-6
HI: *Haemophilus influenzae*
HSV-1: Herpes simplex virus-1
HSV-2: Herpes simplex virus-2
LM: *Listeria monocytogenes*
MP: Mycoplasma pneumoniae
MuV: Mumps virus
NM: Neisseria meningitidis
PCR: Polymerase chain reaction
SP: *Streptococcus pneumoniae*
TB: Tuberculosis
VZV: Varicella-zoster virus

## References

[1] Tavakoli NP, Wang H, Dupuis M, Hull R, Ebel GD, Gilmore EJ, Faust PL (2009). Fatal case of deer tick virus encephalitis. N Engl J Med.

[2] Mailles A, Stahl JP (2009). Infectious encephalitis in France in 2007: a national prospective study. Clin Infect Dis.

[3] Tauber SC, Eiffert H, Kellner S, Lugert R, Bunkowski S, Schutze S, Perske C, Bruck W, Nau R (2014). Fungal encephalitis in human autopsy cases is associated with extensive neuronal damage but only minimal repair. Neuropathol Appl Neurobiol.

[4] Chaudhuri A, Kennedy PG (2002). Diagnosis and treatment of viral encephalitis. Postgrad Med J.

[5] Becerra JC, Sieber R, Martinetti G, Costa ST, Meylan P, Bernasconi E (2013). Infection of the central nervous system caused by varicella zoster virus reactivation: a retrospective case series study. Int J infect Dis.

[6] Misra UK, Tan CT, Kalita J (2008). Viral encephalitis and epilepsy. Epilepsia.

[7] Ihekwaba UK, Kudesia G, McKendrick MW (2008). Clinical features of viral meningitis in adults: significant differences in cerebrospinal fluid findings among herpes simplex virus, varicella zoster virus, and enterovirus infections. Clin Infect Dis.

[8] Vora NM, Holman RC, Mehal JM, Steiner CA, Blanton J, Sejvar J (2014). Burden of encephalitis-associated hospitalizations in the United States, 1998-2010. Neurology.

[9] Granerod J, Ambrose HE, Davies NW, Clewley JP, Walsh AL, Morgan D, Cunningham R, Zuckerman M, Mutton KJ, Solomon T (2010). Causes of encephalitis and differences in their clinical presentations in England: a multicentre, population-based prospective study. Lancet Infect Dis.

[10] Sauerbrei A, Schafler A, Hofmann J, Schacke M, Gruhn B, Wutzler P (2012). Evaluation of three commercial varicella-zoster virus IgG enzyme-linked immunosorbent assays in comparison to the fluorescent-antibody-to-membrane-antigen test. Clin Vaccine Immunol.

[11] Echevarria JM, Casas I, Martinez-Martin P (1997). Infections of the nervous system caused by varicella-zoster virus: a review. Intervirology.

[12] Maple PA, Haedicke J, Quinlivan M, Steinberg SP, Gershon AA, Brown KE, Breuer J (2016). The differences in short- and long-term varicella-zoster virus (VZV) immunoglobulin G levels following varicella vaccination of healthcare workers measured by VZV fluorescent-antibody-to-membrane-antigen assay (FAMA), VZV time-resolved fluorescence immunoassay and a VZV purified glycoprotein enzyme immunoassay. Epidemiol Infect.

[13] Jones D, Blackmon A, Neff CP, Palmer BE, Gilden D, Badani H, Nagel MA (2016). Varicella-zoster virus downregulates programmed death ligand 1 and major histocompatibility complex class I in human brain vascular adventitial fibroblasts, Perineurial cells, and lung fibroblasts. J Virol.

[14] Kennedy PG, Rovnak J, Badani H, Cohrs RJ (2015). A comparison of herpes simplex virus type 1 and varicella-zoster virus latency and reactivation. J General Virol.

[15] Grahn A, Studahl M (2015). Varicella-zoster virus infections of the central nervous system - prognosis, diagnostics and treatment. J Infect.

[16] Lee JE, Lee S, Kim KH, Jang HR, Park YJ, Kang JS, Han SY, Lee SH (2016). A case of transverse myelitis caused by varicella zoster virus in an immunocompetent older patient. Infect Chemother.

[17] De Broucker T, Mailles A, Chabrier S, Morand P, Stahl JP (2012). Acute varicella zoster encephalitis without evidence of primary vasculopathy in a case-series of 20 patients. Microbiol Infect Dis.

[18] Gilden D, Cohrs RJ, Mahalingam R, Nagel MA (2009). Varicella zoster virus vasculopathies: diverse clinical manifestations, laboratory features, pathogenesis, and treatment. Lancet Neurol.

[19] Guo Y, Audry M, Ciancanelli M, Alsina L, Azevedo J, Herman M, Anguiano E, Sancho-Shimizu V, Lorenzo L, Pauwels E (2011). Herpes simplex virus encephalitis in a patient with complete TLR3 deficiency: TLR3 is otherwise redundant in protective immunity. J Exp Med.

[20] Whitley RJ, Kimberlin DW (2005). Herpes simplex encephalitis: children and adolescents. Semin Pediatr Infect Dis.

[21] Zhang H, Cheng H, Wang Q, Zeng X, Chen Y, Yan J, Sun Y, Zhao X, Li W, Gao C (2015). An advanced fragment analysis-based individualized subtype classification of pediatric acute lymphoblastic leukemia. Sci Rep.

[22] Sweeney CJ, Gilden DH (2001). Ramsay hunt syndrome. J Neurol Neurosurg Psychiatry.

[23] Kalghatgi AT, Praharaj AK, Sahni AK, Pradhan D, Kumaravelu S, Prasad PL, Nagendra A (2008). Detection of bacterial pathogens in cerebrospinal fluid using restriction fragment length polymorphism. Med J Armed Forces India.

[24] Nagel MA, Cohrs RJ, Mahalingam R, Wellish MC, Forghani B, Schiller A, Safdieh JE, Kamenkovich E, Ostrow LW, Levy M (2008). The varicella zoster virus vasculopathies: clinical, CSF, imaging, and virologic features. Neurology.

